# A technology‐specific risk analysis of surface guided radiation therapy

**DOI:** 10.1002/acm2.70837

**Published:** 2026-09-30

**Authors:** Victoria Bry, Angelia Tran, Tamara Egan, Eric Ford, Juergen Meyer

**Affiliations:** ^1^ Department of Radiation Oncology, Samuel Oschin Cancer Center Cedars Sinai Medical Center Los Angeles California USA; ^2^ Department of Radiation Oncology, Fred Hutchinson Cancer Center University of Washington Seattle Washington USA

**Keywords:** FMEA, safety and quality, SGRT, surface imaging

## Abstract

**Background:**

Surface Guided Radiation Therapy (SGRT) has become an increasingly important tool that complements x‐ray imaging to improve patient safety for setup, respiratory tracking, and motion monitoring. As SGRT is integrated into clinical workflows, its complexity can introduce potential risk of process‐related errors. These risks may depend on workflow design, system integration, equipment configuration, and technology specific features. As SGRT continues to expand across a wider range of treatment sites and clinical applications, comprehensive evaluation of institutional workflows is recommended to support safe and effective implementation.

**Purpose:**

This study aimed to identify and evaluate safety risks associated with the clinical use of SGRT using Failure Mode and Effects Analysis (FMEA). Emphasis was placed on workflow processes, system integration, and equipment‐specific characteristics within a clinical environment to inform risk mitigation strategies and support safe implementation.

**Methods:**

A multidisciplinary team performed an FMEA of SGRT related procedures and workflows. A process map was developed to define the scope of clinical applications, including tattoo free setup, free‐breathing and deep‐inspiration breath‐hold (DIBH) breast treatments, prone patient positioning and real‐time motion monitoring for stereotactic body radiation therapy (SBRT) patients across body sites. For each process step, team members identified potential failure modes associated with the clinical workflow and environment, including the integration of a C‐RAD SGRT system with Elekta linear accelerators. Scoring was performed according to AAPM TG‐100 guidelines, using severity (S), occurrence (O), and detectability (D) to calculate the Risk Priority Number (RPN). Failure modes were then ranked by RPN, and those with scores greater than or equal to 100 were selected for further analysis and development of mitigation strategies.

**Results:**

Thirty‐eight failure modes were identified, with nine having RPN scores greater than or equal to 100 (*S* = 5–8, *O* = 2–5, and *D* = 5–9). High risk failure modes were most associated with the DIBH workflow, particularly respiratory trace acquisition, respiratory trace configuration, and x‐ray image verification. Additional high risk failure modes were identified in treatment preparation and system quality assurance processes, including manual data import, template selection, calibration, and daily QA procedures. These risks were primarily associated with workflow‐dependent processes involving user interaction and coordination between integrated clinical systems.

**Conclusions:**

This FMEA identified workflow, system integration and equipment related vulnerabilities associated with SGRT implementation. The findings emphasize the importance of risk assessments tailored to specific clinical workflows and treatment environments and support the development of targeted mitigation strategies for safe clinical use.

## 1 INTRODUCTION

Surface guided radiation therapy (SGRT) involves the use of an optical imaging system for patient setup, respiratory motion tracking, and motion monitoring during radiotherapy^1^. The technique is based on registering a reference capture of the patient's surface in treatment position with a live surface capture to assess positional discrepancies. Surveys in the U.S. (2019) and internationally (2022) on SGRT reported that it is most commonly used for initial positioning and intra‐fraction monitoring in breast radiotherapy, with additional applications in stereotactic body radiotherapy (SBRT) and other non‐SBRT sites, including treatment of thorax, abdomen, pelvis, and extremities^2^, ^3^, ^4^. SGRT provides an additional layer of patient monitoring and has demonstrated the ability to improve patient safety^5^ by detecting incorrect positioning, isocenter, and use of immobilization devices. It can act as a real‐time safety net that will notify clinicians when thresholds are surpassed during intra‐fraction motion monitoring and when changes in patient surface anatomy are registered, such as breast inflammation^6^. Al‐Hallaq et al. compared three incident learning systems and reported that approximately 21% of events at the treatment delivery stage could have been prevented with SGRT, particularly those related to incorrect isocenter and accessory placement^5^.

SGRT requires additional training, and integration into the clinic can be complex^3^. Risk assessment of current workflows can identify potential failure modes and guide mitigation strategies to enhance safety^7^. AAPM TG‐302 highlights the importance of risk analysis as part of SGRT implementation and QA program development^6^. Failure Modes and Effects Analysis (FMEA) is a risk assessment methodology commonly used in Radiation Oncology to identify workflow vulnerabilities, prioritize risks, and evaluate processes ranging from routine treatment delivery to high‐risk procedures such as SBRT, and the commissioning of new technologies^8^.

While previous studies have examined risks associated with SGRT systems and specific applications,^9^, ^10^ there remains a need to evaluate risks associated with particular workflow implementations, system integrations, and equipment configurations, as these factors may influence the nature and severity of potential failures. The purpose of this work was to apply FMEA to assess clinical workflows involving a C‐RAD (C‐RAD AB, Uppsala, Sweden) SGRT system in an Elekta (Elekta AB, Stockholm, Sweden) treatment environment, identify potential failure modes, and discuss the most significant risks and mitigation strategies.

## 2 MATERIALS & METHODS

### 2.1 Clinical workflow description

A multidisciplinary team of users with 3–8 years of experience with SGRT (3 medical physicists and 2 radiation therapists) provided input on the use of SGRT to assess clinical workflows. The team met as a whole and in smaller groups to discuss and expand upon an existing published general process map of SGRT^11^. The goal was to create an institution‐specific process map that consisted of the SGRT system installed in the CT simulation (Sentinel®) and treatment rooms (Catalyst+®). Clinical equipment included a CT scanner (GE Medical Systems Inc., Chicago, IL) and Elekta Versa HD and Infinity Linacs. Figure 1 illustrates the adjusted process map and clinical workflow, developed at two clinics with a total of three linacs. At the time of this study, SGRT was primarily used at our institution for positioning and motion management for breast/chest, thoracic spine, thoracic and prone pelvis treatments, and extensively for DIBH in left sided breast treatments and real‐time monitoring of all SBRT patients. Tattoo‐free setup was implemented for all treatment sites of the upper torso, and a three‐point laser setup was used for initial setup followed by SGRT for motion monitoring of pelvis treatments.

**FIGURE 1:**
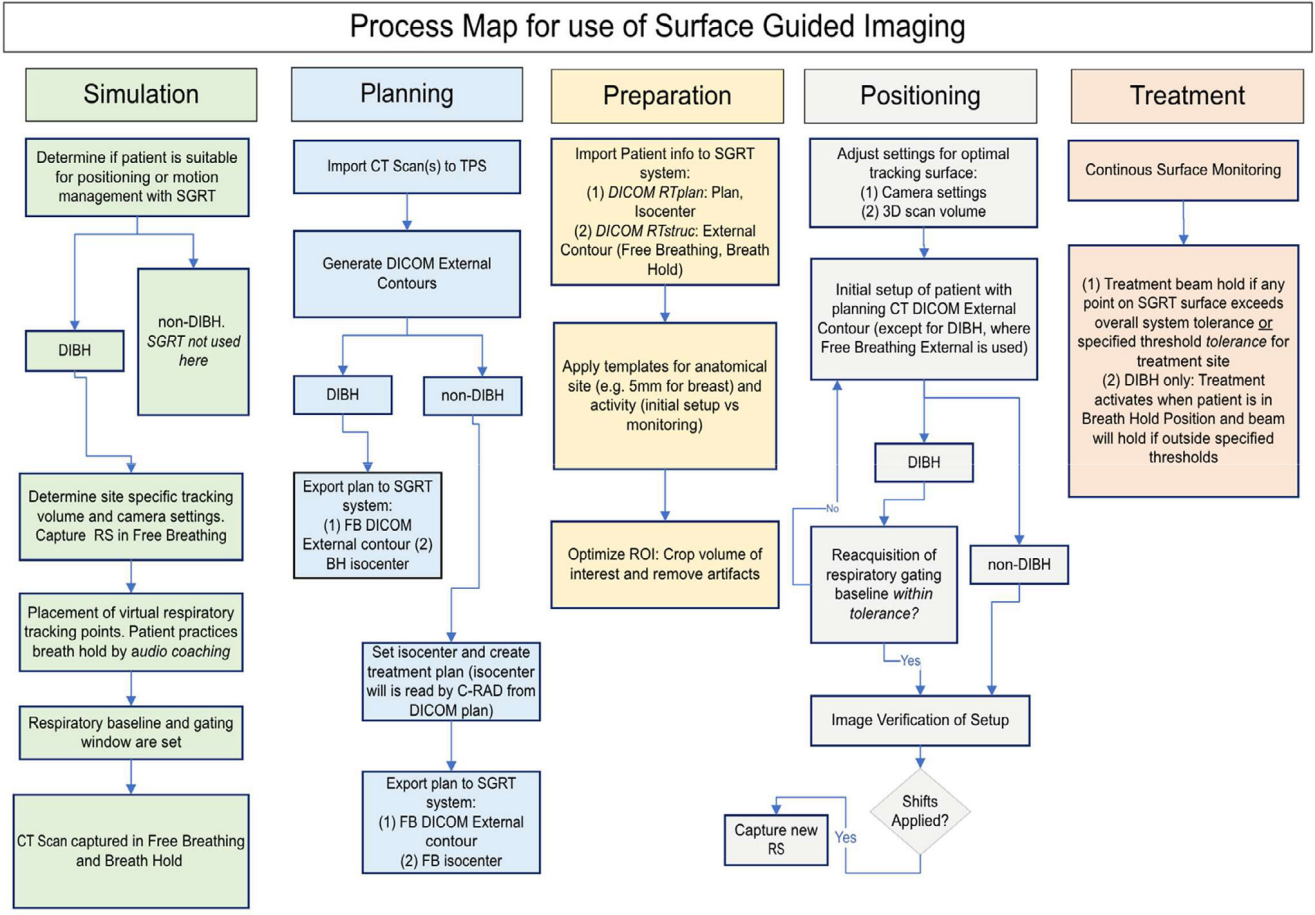
Process Map. SGRT process map for DIBH and non‐DIBH patients from Simulation to Treatment Delivery.

At the time of simulation, the patient's setup approach is determined based on treatment site and clinical protocol, including whether a tattoo‐based or tattoo‐free setup will be used, with or without the use of SGRT. For a DIBH simulation, CT scans are acquired in both free breathing (FB) and breath‐hold (BH) conditions. Patients are considered eligible if they can consistently maintain a breath‐hold for at least 20 seconds.

They will practice BH through audio coaching, and a radiation therapist will set a *primary* virtual respiratory tracking point with the C‐RAD SGRT system on the midline of a patient's chest below the Xiphoid process,^12^ which allows for tracking of respiratory motion. The respiratory baseline is defined by the system as the lowest point of the breathing trace during the first 10 seconds of FB acquisition. The breath‐hold gating window is then established around a target breath‐hold level corresponding to approximately 80% of the patient's maximum deep inspiration. For all other treatment sites and workflows, SGRT is not utilized during CT simulation.

In the process map, treatment planning involves generation of a reference surface from the external body contour delineated on the free‐breathing CT dataset, referred to as the *DICOM external*, within the treatment planning systm (TPS) (RayStation, RaySearch Inc., Stockholm, Sweden). For plan preparation, the DICOM‐RT plan and DICOM external structure are exported from the TPS to a file directory and then manually copied to the C‐RAD import directory for import into the SGRT system. Direct transfer from the TPS was not available because the C RAD system did not support a DICOM server connection for automated communication with the TPS. In addition, local IT infrastructure did not permit direct export of DICOM files from the TPS to the C RAD incoming directory. As a result, patient data transfer was performed manually as part of the clinical workflow. For DIBH patients, the DICOM external structure provides the reference surface used for patient positioning and motion monitoring, while the DICOM‐RT plan provides treatment delivery information, including beam geometry, isocenter information, and couch rotations. Site specific templates with empirically derived SGRT tolerances are then assigned.

Prior to treatment, a virtual simulation (Vsim) or dry run of the patient's treatment is completed to ensure safe delivery. This process includes patient specific adjustment of SGRT camera settings (integration time and signal gain) for patient specific surface characteristics and selection of VOI (Volume of Interest) or tracking volume. Initial patient setup is performed using the DICOM external reference surface and verified using x‐ray image verification. For VMAT treatments, a kV cone‐beam CT is acquired and registered to the planning CT using internal anatomical landmarks (bony or soft tissue), whereas for 3D tangent breast treatments, orthogonal portal images are acquired and compared with reference DRRs (digitally reconstructed radiographs) generated from the planning CT. Following any couch shifts determined from x‐ray image registration, a new SGRT reference surface is acquired to ensure agreement between the external surface monitored by SGRT and the verified internal anatomy.

For real time motion monitoring, C‐RAD is integrated with the linac's control system via the Elekta Response ® interface and automatically triggers a ‘beam off’ the treatment beam under the following scenarios: (1) isocenter shift distance between live and reference images exceeds site‐specific tolerance, (2) DIBH patient moves outside the respiratory tolerance window or (3) any point between the two surfaces exceeds a predefined tolerance, even if isocenter is within tolerance and the respiratory signal is within the gating window.

At the time of this study, breast radiotherapy was one of the primary treatment sites at our institution, and DIBH was routinely used for eligible patients. As a result, DIBH related workflows constituted a substantial component of SGRT clinical practice and were well represented in the process map and FMEA analysis. Workflow frequency was not quantitatively assessed and was not incorporated into the FMEA scoring.

### 2.2 FMEA process

A team leader facilitated a step‐by‐step review of the clinical process described above with all members of the team, during which known failures, their causes, and potential effects were discussed. Failure modes were scored according to the guidance of AAPM TG‐100^8^ using a 1–10 scale for occurrence (O), detectability (D) and severity (S), with the risk priority number (RPN = S*O*D)^6^ calculated for each failure mode. Initial scoring was performed independently by two physicists as a structured expert assessment of perceived workflow risk. Differences in scoring were not formally recorded or analyzed as a measure of inter‐rater variability. Instead, discrepancies were reviewed through discussion with the team until agreement was reached and consensus scores were assigned for the final analysis. Failure modes with RPN scores greater than or equal to 100 were selected as a practical threshold for further evaluation. This threshold was chosen to focus the analysis on the highest‐ranked failure modes while maintaining a manageable number of events for detailed review and development of mitigation strategies.

## 3 RESULTS

A total of 38 failure modes (Table S1) were identified (RPN scores: 4–210) and nine of these (24% of all failure modes) had an RPN score equal to or greater than 100 (listed in Table 1 *)*. Out of all the failure modes, sixteen were associated with the DIBH workflow, nine with treatment preparation, and three with SGRT QA/calibration. The absolute difference (**Δ)** in RPN between scorers ranged from 0 to 66 for top failure modes. One received identical RPN scores from both scorers (ΔRPN = 0), while the remaining failure modes demonstrated varying degrees of disagreement. For seven of these eight failure modes, the same scorer's ratings produced a higher calculated RPN, except for failure mode 3 (initial acquisition of the respiratory baseline), where the pattern reversed. Five of the highest‐risk errors occurred in process steps specific to the DIBH workflow and four of these were related to respiratory trace acquisition or configuration.

**TABLE 1:** High‐priority failure modes identified by FMEA (RPN ≥ 100).

Rank	Workflow	Process	Potential Failure Modes	Potential Cause	Potential Effect	Action Items	Avg O	Avg S	Avg D	Avg RPN	Δ RPN (RPN Scorer 1 ‐ RPN Scorer 2):
1	Positioning (DIBH specific)	Reacquisition of respiratory baseline	Newly acquired baseline is out of tolerance and overridden, causing large differences in lung volume for the breath‐hold position, (e.g. patient arching back or altered posture)	Not following workflow; inadequate coaching; patient fatigue; baseline re‐acquired in non‐reproducible position.	Treatment not delivered accurately; internal anatomy may not correspond to planned location of anatomy.	✓ *Re‐setup patient* ✓ *Improve coaching to return to reproducible baseline* ✓ *Repeat breath‐hold image verification* ✓ *Add review of baseline reacquisitions to weekly physics checks*.	5.0	7.0	6.0	**210.0**	**44**
2	Positioning (DIBH specific)	Breath Hold Image Verification	Patient exits respiratory gating window during kV‐CBCT acquisition, resulting in image obtained with incorrect ‘breath hold' amplitude	*Lack of interlocks between SGRT and kV‐imaging*. Not following workflow. Inadequate coaching of patient.	Incorrect patient setup; image may not represent intended breath‐hold anatomy; Treatment not delivered accurately	✓ *Reinforce protocol to require therapists to watch gating window during image acquisition*. ✓ *Verify breath hold anatomy aligns with simulation* ✓ *Repeat breath‐hold image verification*	3.0	7.5	9.0	**202.5**	**27**
3	Simulation (DIBH specific)	Initial acquisition of respiratory baseline	Inappropriate acquisition of respiratory baseline during initial tracking period (determined in first 10 seconds)	Not following workflow. Inadequate coaching of patient or irregular breathing during baseline acquisition.	Respiratory gating may not be reproducible at treatment. Treatment delay and/or potential repeat simulation or free‐breathing treatment.	✓ *Provide regular therapist training* ✓ *Standardize coaching instructions* ✓ *Confirm reproducible breath‐hold trace before CT acquisition*.	5.0	7.5	5.0	**187.5**	**50**
4	Treatment	Motion Monitoring	Inappropriate override of patient motion ‘out of tolerance’	Not following workflow	Treatment not delivered accurately	✓ *Provide clear expectations for when overrides are acceptable* ✓ *Review SGRT overrides during weekly physics checks*.	4.0	6.5	7.0	**182.0**	**28**
5	Simulation (DIBH specific)	Setting of respiratory gating window with audio coaching	Variable breath hold depth during imaging resulting in CT not acquired at desired amplitude.	Not following workflow; inadequate coaching of patient or inconsistent breath‐hold performance.	Non‐optimal sparing of the heart; reduced reproducibility between simulation and treatment.	✓ *Improved patient coaching* ✓ *Physics review of respiratory window and manual adjustment of breathing window when appropriate*.	2.5	6.5	8.5	**138.1**	**60**
6	Preparation	Manual selection of templates for motion management	Incorrect site‐specific threshold template selected.	Not following workflow or manual selection error.	Non optimal tx delivery; tolerances may be too loose or restrictive, leading to unnecessary treatment interruptions or undetected motion.	✓ *Require a second verification during treatment preparation*.	3.0	5.0	8.5	**127.5**	**66**
7	Preparation (DIBH specific)	Import of patient data into SGRT system	Incorrect respiratory trace selected for treatment	Not following workflow or manual selection error. (e.g. confusion from multiple traces available)	Non‐optimal treatment delivery; breath‐hold gating may not correspond to planned simulation of breath hold.	✓ *Standardize respiratory trace naming or document intended trace at simulation* ✓ *Require a second verification during preparation*.	2.0	7.5	8.0	**120.0**	**16**
8	QA	Daily QA	Daily QA is performed with phantom in incorrect position	QA setup error	Systematic SGRT offset; inaccurate surface tracking	✓ *Standardize QA setup instructions* ✓ *Trend daily QA results*	3.0	8.0	5.0	**120.0**	**0**
9	Preparation	Import of patient data into SGRT system	Incorrect patient surface imported	Not following workflow or manual selection error (e.g. confusion from multiple plans available)	Breath‐hold or setup reference surface does not correspond to the intended treatment plan; treatment may not be delivered accurately.	✓ *Require a second verification during treatment preparation*	2.5	8.0	5.0	**100.0**	**65**

The most severe risks (S > 7) were associated with patient positioning, simulation, preparation and QA such as setting of respiratory traces, breath‐hold image verification, incorrect import of patient information into the SGRT system, and incorrect daily QA/calibration. The likelihood that each of these failures would occur were most often ranked with lower scores for occurrence (O≤3), except for failures associated with setting of respiratory trace and inappropriate override patient motion ‘out of tolerance’ (4≤ O ≤5). Detectability of all failure modes were scored with medium (5≤ D < 7) to high (D≥7) scores, highlighting concerns that these may be challenging to detect.

To understand how these failures manifested within our practice, we examined some key process steps in our clinical workflow.

✓ During CT simulation, we identified the potential to acquire a non‐optimal initial respiratory baseline (Figure 2) or gating window due to system specific function and insufficient coaching. An incorrect respiratory trace may result in coaching the patient at treatment to take a more shallow or deeper breath than what was originally planned at simulation, thus preventing a reproducible respiratory gating for treatment and inconsistencies of internal anatomy.
✓ CBCT image verification for breath‐hold patients is important for treatment setup to ensure reproducibility of internal anatomy and gating, however, this step is vulnerable to inter‐fractional changes. If a patient moves outside breath‐hold tolerance during the imaging process, there are no interlocks between the SGRT and kV‐CBCT system to identify this, which could result in images acquired at variable lung volumes and chest amplitudes. After application of positional shift corrections, it may also be necessary to capture a new reference surface (different from DICOM) and reacquire a respiratory baseline to account for the patient's anatomy and positioning on the day of treatment, rather than relying on the original DICOM reference surface^11^. Clinical tolerances for reacquisition of respiratory baselines should be carefully considered (Figure 3) as changes in patient positioning or breath‐hold level may introduce discrepancies between the planned and treatment anatomy. Such discrepancies could affect target localization and alter the dose delivered to nearby organs at risk, including the heart.

**FIGURE 2:**
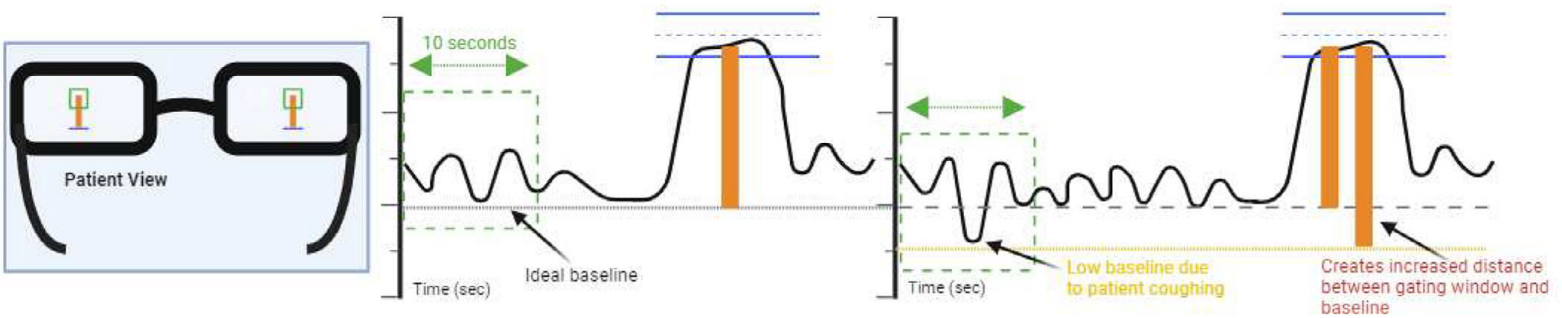
Initial acquisition of respiratory baseline setting. The innate nature of the respiratory trace acquires a baseline within the first ten seconds of recording a patient's breathing and faulty acquisition occur if within ill timing such as patient motion (e.g. coughing, motion from patient discomfort). If identified in plan preparation, a physicist can make an adjustment. Created in BioRender. Bry, V. (2026) https://BioRender.com/hljmxfk.

**FIGURE 3:**
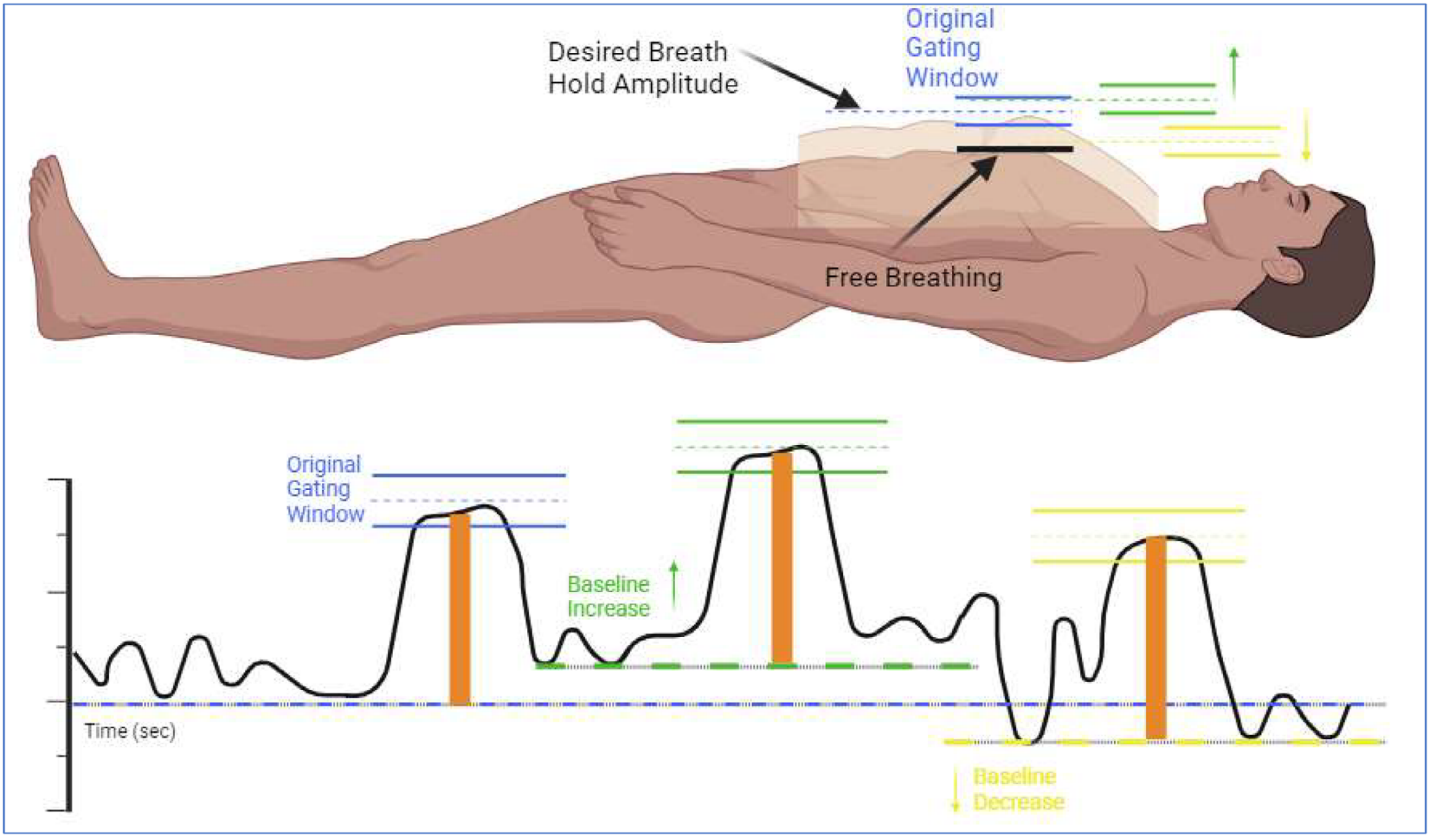
Reacquisition of baseline settings that exceed clinical tolerance. If gating baseline is shifted an amount that exceeds tolerance, it is possible that the patient has shifted anatomical position (ex. patient arching back) and as a result, there could be discrepancies in lung volume. (Created in BioRender. Bry, V. (2026) https://BioRender.com/ugfc465).

Additional failure modes were identified during treatment preparation and quality assurance, including incorrect import of patient information into the SGRT system, inappropriate manual selection of site‐specific templates or tolerances, and error in daily QA or system calibration. These failure modes were predominantly associated with manual workflow processes requiring user input, verification, and coordination between clinical systems, highlighting the potential for human error during SGRT configuration and preparation, and quality assurance activities.

Mitigation strategies implemented for failure modes in Table 1 include both patient specific corrective actions and workflow changes. For failure modes involving breath‐hold verification, corrective actions included re‐setup of a patient, additional breath‐hold coaching and repeat image verification, paired with prospective workflow changes such as standardized coaching instructions and in‐advance respiratory training^13^, ^14^, ^15^ and home practice, regular therapist training, and reinforced protocols requiring direct therapist observation during image acquisition. For failure modes involving manually entered data during treatment preparation, a second verification step was added. Review of motion‐monitoring overrides and respiratory baseline changes was incorporated into weekly physics QA checks.

## 4 DISCUSSION

A risk analysis of clinical workflows involving a C‐RAD SGRT system integrated with Elekta linear accelerators identified 38 failure modes, nine of which had RPN values greater than or equal to 100. The highest risk failure modes were associated with workflow steps requiring manual decision making, user verification, and coordination between multiple clinical systems, particularly for respiratory motion management, breath‐hold verification, treatment preparation, and quality assurance. These findings highlight the importance of standardized procedures, training, and independent verification practices to support safe SGRT implementation. While many of the identified failure modes are broadly applicable to SGRT, differences in system integration, equipment configuration, imaging platforms, and clinical procedures may introduce workflow specific vulnerabilities. A multidisciplinary evaluation of local clinical workflows can help identify potential failure modes and inform mitigation strategies tailored to the treatment environment.

Patient data was manually transferred and imported into the SGRT system because at the time of this study, the SGRT system did not support direct DICOM communication with the TPS. In addition, institutional IT constraints prevented direct export of DICOM files to the SGRT import directory. This process provides for an independent verification step to confirm the correct patient, treatment plan, reference surface, respiratory trace, and isocenter. This manual process also introduces opportunities for user error, including incorrect selection of site‐specific templates, respiratory traces, or reference surfaces. While increased automation may reduce the risk of manual errors, imported patient information should still be independently verified. Automated workflows may also introduce different failure modes related to data transfer, software integration, or communication between connected systems^16^.

The highest‐ranked failure modes were not uniformly attributed to the SGRT vendor, but rather reflected platform‐specific characteristics and system integration within the clinical workflow. Some failure modes were directly influenced by vendor‐specific implementation details. For example, respiratory baseline acquisition depends on how the system establishes and calculates the FB baseline signal, which may vary between platforms and influence respiratory trace configuration and reproducibility. Therapist response to patient “out‐of‐tolerance” notifications may be influenced by the design of the user interface, the ease with which these notifications can be overridden, and institutional clinical procedures. Similarly, the absence of an interlock between SGRT and kV‐CBCT reflects the level of system integration within the treatment environment. In this case, the inability of the SGRT system to detect whether a patient moves outside the breath‐hold tolerance during CBCT acquisition is not solely attributable to the SGRT platform but also reflects the absence of an interface that enables communication between the linac imaging system and the SGRT system. Therefore, these findings should be interpreted within the context of the specific platform, system integration, and clinical workflow evaluated in this study, rather than as risks exclusively attributable to a particular SGRT vendor.

As an example, one failure mode identified during acceptance testing was that the C‐RAD system considered field identification and table rotation for matching without verification of isocenter coordinates between the OIS (Mosaiq) and the SGRT system. During testing, it was possible to achieve a successful match based solely on the field identifier despite discrepancies in coordinate information, without triggering an error message. This observation highlights the importance of comprehensive commissioning and verification of system interfaces to identify inconsistencies in data transfer and system communication. To mitigate this risk, manual verification of imported SGRT data is performed by the physics team to confirm agreement of patient information, treatment plan parameters, and isocenter coordinates between systems.

Previous risk analysis of SGRT have evaluated a general workflow^11^ and workflows specific for DIBH^9^ and cranial radiosurgery^10^. The European guidelines^11^ categorized general SGRT failures as ‘workflow, insufficient training’ and ‘commissioning, calibration and QA.’ These include incorrect use of the reference surface, isocenter, protocol, or volume of interest (VOI), inadequate commissioning/QA related to calibration, misinterpretation of the coordinate system, or gantry occlusion^11^. They provide potential solutions to these failures and highlight the importance of using IGRT verification and establishing department protocols. FMEA studies on DIBH^9^ and cranial radiosurgery^10^ utilized the AlignRT® (VisionRT, London, UK) SGRT system and reinforced our findings. For linac‐based SRS with SGRT, the most severe and common occurrences were linked to incorrect calibration, inadequate QA and inability to track the surface due to signal obstruction. Suggested mitigations included day‐of‐treatment QA verification, independent second checks, and continuous physics oversight^10^.

A risk analysis on DIBH workflows using SGRT^9^ linked most severe failures to operator error and thus emphasized the importance of comprehensive staff training and highlights the benefit of the use of checklists or task‐specific protocols to mitigate failures^9^. One failure mode that could result in a treatment not being delivered correctly was related to patient motion during capture of a new surface reference that is outside treatment position, due to movement during the imaging process. To mitigate this, a protocol was implemented so for at least one therapist to watch the patient while another captures an external reference and to validate alignment of anatomy with a verification port after all shifts.

Uncertainty in BH techniques arises from both interfractional variability between treatment sessions and intrafractional variability within a single treatment driven by differences in breathing pattern (chest vs abdominal), patient positioning, internal organ motion, or fatigue^17^. Variation in breathing patterns can lead to inconsistent BH volumes, resulting in inter‐fractional shifts in tumor position and geometric accuracy^18^. This uncertainty can be difficult to detect but can be analyzed during the planning process with multiple BH scans to delineate patient specific margins^19^ or use of the same BH monitoring approach or vendor technology between simulation and treatment to provide maximum reproducibility and uniformity between planning and treatment.

The dosimetric importance of these uncertainties has been further investigated by Kügele et al. (2017)^20^ and Ranganathan et al. (2026)^21^. These studies showed that small deviations for an externally monitored DIBH position may not accurately reflect internal target and OAR motion. Both studies showed that deviations can result in clinically meaningful dosimetric consequences, and that breath‐hold undershoot was associated with reduced PTV coverage.

Kügele et al. (2017) evaluated intrafractional variability between breath‐holds using SGRT by applying rigid isocenter shifts to the planned dose distribution. Reproducibility was generally acceptable, with a median score of ∼1 mm, however, an occasional shift up to 5 mm affected PTV coverage and OAR dose. The largest effects occurred when the treatment field edge was near the heart or left anterior descending (LAD) structure so that small shifts resulted in these structures entering or exiting the treatment field. Authors of this study also emphasized the need to establish tolerances not only for the external surrogate (e.g. VOI of xiphoid process) but also for the estimated isocenter position^20^. In contrast, Ranganathan et al. (2026) used deformable image registration to model DIBH deviations within a clinically accepted tolerance (± 5 mm) in 20 patients with left‐sided breast cancer. By using a more realistic internal deformation, they showed that dose effects were highly patient and technique specific. 3D conformal plans were generally more robust with respect to target coverage, but pseudo‐skin‐flash plans provided comparable coverage during breath‐hold overshoot^14^. These findings highlight the need for workflow‐specific strategies that maintain BH reproducibility and verify that the external signal reflects the intended internal target and OAR positions. Consistent with this need, the European Society for Radiotherapy and Oncology (ESTRO) and its Advisory Committee for Radiation Oncology Practice (ACROP) recommends to define a minimum achievable BH duration to ensure sufficient time for imaging acquisition, particularly for workflows without an interlock between the BH monitoring system and the linac, where the radiation therapists (RTT) are required to manually beam on and off^17^.

At treatment time, lung volume filling differences can be compared using x‐ray imaging, and additional insight into anterior‐posterior surface differences can be provided by overlaying external body and lung contours. Another factor related to patient coaching is that BH reproducibility is dependent on the feedback methods provided to the patient. Audio and/or visual techniques are commonly utilized to guide patients to perform a reproducible breath amplitude for DIBH treatments^22^ and prior studies suggest that audio‐visual feedback may improve reproducibility compared to audio coaching alone^23^. At the time of this analysis only audio coaching was implemented at CT simulation, which may contribute to variability in breath‐hold imaging and amplitude reproducibility. AAPM TG302 suggests that if breath‐hold varies for a DIBH specific patient, despite coaching, then reproducibility should be assessed or the treatment plan may need to be adapted^6^.

It is important to acknowledge several limitations of this study. First, the FMEA was performed at a single institution within a specific environment, therefore identified failure modes may not be directly generalizable to other vendors' workflows. The analysis focused on workflow‐related risks and did not evaluate patient‐specific factors such as body habitus, skin tone, tattoos, couch sag, or surface deformation over the course of treatment which may affect tracking quality and accuracy. Second, a consensus scoring approach was performed by two medical physicists based on clinical experience and expert judgment, resulting in RPN scores that are inherently subjective, which may vary among institutions. Third, failure modes with RPN scores ≥100 were selected for analysis. However, institutions should also consider both RPN and individual severity ratings as these may be clinically relevant. Finally, the identified failure modes were not correlated with actual incident reports and reflect perceived risk. Future studies incorporating SGRT and incident learning system datasets would benefit from validating the frequency and dosimetric impact of these failures to refine their relative importance. Prospective implementation of the proposed mitigation strategies followed by a repeat FMEA would be helpful to assess post‐mitigation RPN scores and quantify the effectiveness of these interventions in reducing workflow risk.

Overall, it is important to understand and quantify the BH related uncertainties within each institution and incorporate strategies to account for this in margin design and workflows and continuously assess protocols to ensure accurate and reproducible. As the role of SGRT is evolving to cover a wider range of applications, a comprehensive evaluation of a variety of workflows is warranted to identify new workflow related, platform specific, and system integration related failure modes. Our analysis builds on prior studies by emphasizing vulnerabilities unique to gated workflows and role the physics team plays to mitigate these risks. Risks of failure can be reduced through standardized staff training, protocols and checklists, QA, and monitoring strategies.

## 5 CONCLUSIONS

This FMEA evaluated the C‑RAD SGRT system in an Elekta environment and identified 38 failure modes, including nine high‑risk modes (RPN ≥100). The highest‑risk issues occurred in the DIBH workflow. The main vulnerabilities involved system‑specific respiratory baseline determination, coaching variability, lack of interlocks between SGRT and CBCT imaging, and manual data handling tasks. These findings indicate that workflow‑dependent processes and integration gaps were the primary drivers of SGRT risk. These results directly informed the mitigation strategies, including standardized coaching, second‑check verification of manual imports, enhanced therapist training, and weekly physics review of overrides and respiratory baseline changes. As SGRT expands to additional treatment sites and more complex applications, our findings highlight the need for ongoing, workflow‑specific risk assessment, particularly for gated techniques where reproducibility and system coordination are critical. The vulnerabilities underscore the importance of robust QA procedures, standardized protocols, and multidisciplinary oversight to ensure safe and effective SGRT implementation. They also emphasize the need for vendors to collaborate more closely to improve system interfaces and communication pathways, reducing integration‑related failure modes. This work adds to the growing literature advocating proactive safety practices in radiation oncology and supports the development of standardized approaches tailored to specific technologies and institutional workflows.

## AUTHOR CONTRIBUTIONS

**Victoria Bry**: Methodology; formal analysis; investigation; data curation; visualization; writing—original draft; writing—review and editing; project administration. **Angelia Tran**: Methodology; investigation; supervision; writing—review and editing. **Tamara Egan**: Methodology; investigation; writing—review and editing. **Eric Ford**: Conceptualization; methodology; investigation; supervision; writing—review and editing. **Juergen Meyer**: Conceptualization; methodology; investigation; supervision; writing—original draft; writing—review and editing.

## CONFLICT OF INTEREST STATEMENT

The authors declare no conflicts of interest.

## Supporting information




Supporting Information

## Data Availability

The data supporting the findings of this study are included within the article and its supplementary material.

## References

[1] Hoisak JDP , Pawlicki T . The role of optical surface imaging systems in radiation therapy. Semin Radiat Oncol. 2018;28:185‐193. doi:10.1016/j.semradonc.2018.02.003 29933878

[2] Padilla L , Havnen‐Smith A , Cerviño L , Al‐Hallaq HA . A survey of surface imaging use in radiation oncology in the United States. J Appl Clin Med Phys. 2019;20:70‐77. doi:10.1002/acm2.12762 PMC6909172 31743588

[3] Batista V , Gober M , Moura F , et al. Surface guided radiation therapy: an international survey on current clinical practice. Tech Innov Patient Support Radiat Oncol. 2022;22:1‐8. doi:10.1016/j.tipsro.2022.03.003 PMC8984757 35402740

[4] Meyer J , Tran A , Ma TM , et al. A hybrid IGRT workflow using SGRT and CBCT for prostate SBRT: feasibility, efficiency, and safety. J Appl Clin Med Phys. 2025;26:e70339. doi:10.1002/acm2.70339 PMC12593551 41201189

[5] Al‐Hallaq H , Batista V , Kügele M , Ford E , Viscariello N , Meyer J . The role of surface‐guided radiation therapy for improving patient safety. Radiother Oncol. 2021;163:229‐236. doi:10.1016/j.radonc.2021.08.008 34453955

[6] Al‐Hallaq HA , Cerviño L , Gutierrez AN , et al. AAPM task group report 302: surface‐guided radiotherapy. Med Phys. 2022;49:e82‐e112. doi:10.1002/mp.15532 PMC9314008 35179229

[7] Batista V , Meyer J , Kügele M , Al‐Hallaq H . Clinical paradigms and challenges in surface guided radiation therapy: where do we go from here?. Radiother Oncol. 2020;153:34‐42. doi:10.1016/j.radonc.2020.09.041 32987044

[8] Huq MS , Fraass BA , Dunscombe PB , et al. The report of Task Group 100 of the AAPM: application of risk analysis methods to radiation therapy quality management. Med Phys. 2016;43:4209‐4262. doi:10.1118/1.4947547 PMC4985013 27370140

[9] Bright M , Foster RD , Hampton CJ , Ruiz J , Moeller B . Failure modes and effects analysis for surface‐guided DIBH breast radiotherapy. J Appl Clin Med Phys. 2022;23:e13541. doi:10.1002/acm2.13541 PMC8992938 35112445

[10] Manger RP , Paxton AB , Pawlicki T , Kim G‐Y . Failure mode and effects analysis and fault tree analysis of surface image guided cranial radiosurgery. Med Phys. 2015;42:2449‐2461. doi:10.1118/1.4918319 25979038

[11] Freislederer P , Batista V , Öllers M , et al. ESTRO‐ACROP guideline on surface guided radiation therapy. Radiother Oncol. 2022;173:188‐196. doi:10.1016/j.radonc.2022.05.026 35661677

[12] Rasmussen K , Bry V , Papanikolaou N . Technical overview and features of the C‐RAD catalyst and sentinel 4D‐CT systems. In: Hoisak JDP , Paxton A , Waghorn BJ & Pawlicki T , eds. Surface Guided Radiation Therapy. CRC Press; 2020. doi:10.1201/978042948940210.1201/9780429489402-3

[13] Kim A , Kalet AM , Cao N , et al. Effects of Preparatory Coaching and Home Practice for Deep Inspiration Breath Hold on Cardiac Dose for Left Breast Radiation Therapy. Clin Oncol. 2018;30:571‐577. doi:10.1016/j.clon.2018.04.009 29773446

[14] Kalet AM , Kim A , Hippe DS , et al. The dosimetric benefit of in‐advance respiratory training for deep inspiration breath holding is realized during daily treatment in left breast radiotherapy: a comparative retrospective study of serial surface motion tracking. J Med Imaging Radiat Oncol. 2021;65:354‐364. doi:10.1111/1754-9485.13181 PMC8252041 33932102

[15] Mayr NA , Borm KJ , Kalet AM , et al. Reducing cardiac radiation dose from breast cancer radiation therapy with breath hold training and cognitive behavioral therapy. Top Magn Reson Imaging TMRI. 2020;29:135‐148. doi:10.1097/RMR.0000000000000241 32568976

[16] Nealon KA , Balter PA , Douglas RJ , et al. Using Failure Mode and Effects Analysis to Evaluate Risk in the Clinical Adoption of Automated Contouring and Treatment Planning Tools. Pract Radiat Oncol. 2022;12:e344‐e353. doi:10.1016/j.prro.2022.01.003 35305941

[17] Aznar MC , Carrasco de Fez P , Corradini S , et al. ESTRO‐ACROP guideline: Recommendations on Implementation of breath‐hold techniques in radiotherapy. Radiother Oncol. 2023;185:109734. doi:10.1016/j.radonc.2023.109734 37301263

[18] Glide‐Hurst CK , Gopan E , Hugo GD . Anatomical and Pathological Variability during Radiation Therapy for a Hybrid Active Breath Hold Gating Technique. Int J Radiat Oncol Biol Phys. 2010;77:910‐917. doi:10.1016/j.ijrobp.2009.09.080 PMC2956181 20510201

[19] Mast M , Kouwenhoven E , Roos J , et al. Two years’ experience with inspiration breath‐hold in liver SBRT. Tech Innov Patient Support Radiat Oncol. 2018;7:1‐5. doi:10.1016/j.tipsro.2018.04.001 PMC7033777 32095574

[20] Kügele M , Edvardsson A , Berg L , Alkner S , Andersson Ljus C , Ceberg S . Dosimetric effects of intrafractional isocenter variation during deep inspiration breath‐hold for breast cancer patients using surface‐guided radiotherapy. J Appl Clin Med Phys. 2018;19:25‐38. 10.1002/acm2.12214 PMC5768000 29139223

[21] Ranganathan VT , Gouthro A , Stevens T . Using deformable registration to assess dosimetric impact of variability in deep inspiration breath hold (DIBH) levels for left breast treatment. J Appl Clin Med Phys. 2026;27:e70541. doi:10.1002/acm2.70541 PMC13067201 41960694

[22] Yamauchi R , Mizuno N , Itazawa T , Masuda T , Akiyama S , Kawamori J . Assessment of visual feedback system for reproducibility of voluntary deep inspiration breath hold in left‐sided breast radiotherapy. J Med Imaging Radiat Sci. 2021;52:544‐551. doi:10.1016/j.jmir.2021.08.018 34538757

[23] Goossens S , Senny F , Lee JA , Janssens G , Geets X . Assessment of tumor motion reproducibility with audio‐visual coaching through successive 4D CT sessions. J Appl Clin Med Phys. 2014;15:4332. doi:10.1120/jacmp.v15i1.4332 PMC5711223 24423834

