# Supplementary material for: A technology‐specific risk analysis of surface guided radiation therapy
Source: J Appl Clin Med Phys. 2026 Sep 30;27(10):e70837. doi: 10.1002/acm2.70837 (PMC13626861; doi:10.1002/acm2.70837)
Supplement: Supplementary file 1 [file ACM2-27-e70837-s001.pdf]

Supplementary Table S1. Potential Failure Modes, Causes and Effects Identified

Phase	Process Step	Potential Failure Mode	Potential Cause	Potential Effect	Avg O	Avg S	Avg D	Avg RPN	Rank
SIMULATION	Quality Assurance (QA)	Incorrect calibration of SGRT system in CT Simulation (C-RAD Sentinel)	Not following workflow	Patient setup performed incorrectly; treatment not delivered accurately	2.5	7.5	2.5	46.88	18
	Determine if patient is suitable candidate for DIBH	Inappropriate patient selection for DIBH	Inadequate assessment of the patient's ability to understand instructions, maintain a reproducible breath hold, or tolerate DIBH (e.g. compromised lung function) or other patient-specific factors	Inability to perform reproducible DIBH at treatment, resulting in increased treatment time and potential replanning to FB	4.5	3.5	1.5	23.63	26
		DIBH selected despite no clinically meaningful dosimetric benefit	Inadequate patient-specific assessment of the expected benefit of DIBH	Unnecessary treatment complexity and increased simulation or treatment time	2.5	1.5	1.5	5.63	37
	**DIBH Specific:**								
	Determine scan volume and camera settings	Incomplete reference surface captured in ct simulation (C-RAD Sentinel)	Inadequate camera settings or obstruction of the tracking surface by immobilization devices, blankets, or other objects	Reduced surface coverage, potentially resulting in inaccurate or less reproducible initial setup	1.5	7	2	21	27
	Determine respiratory tracking points, baseline and gating amplitude *Use of audio and visual coaching*	Inadequate placement of respiratory tracking points or region of interest	Tracking point positioned over an area that does not reliably represent reproducible respiratory motion. Differences in camera geometry, patient anatomy, or couch position between Sentinel™ and Catalyst+HD may also affect tracking	Unable to accurately or reproducibly monitor respiratory motion during treatment. Potential treatment delay	2.5	4.5	4	45	19
		Inappropriate acquisition of respiratory baseline during initial tracking period (determined in first 10 seconds)	Not following workflow. Inadequate coaching of patient or irregular breathing during baseline acquisition.	Respiratory gating may not be reproducible at treatment. Treatment delay and/or potential repeat simulation or free-breathing treatment.	5	7.5	5	187.5	3
	Capture of FB and BH CT scan	Sentinel reference surface acquired when the patient is not in free breathing	Not following workflow. Incorrect patient instruction or reference-surface acquisition at the wrong point in the respiratory cycle	Reference surface does not accurately represent intended free-breathing position, potentially reducing setup accuracy or reproducibility	3	1	3	9	35
		FB CT and BH CT acquired with different CT origins	Patient or couch movement between scans, or inadvertent resetting of the couch/table coordinates	Incorrect spatial relationship between FB and BH datasets, potentially affecting image registration, planning, or setup; Treatment not delivered accurately	2.5	7.5	4.5	84.38	12

Phase	Process Step	Potential Failure Mode	Potential Cause	Potential Effect	Avg O	Avg S	Avg D	Avg RPN	Rank
		Variable breath hold depth during imaging resulting in CT not acquired at desired amplitude.	Not following workflow; inadequate coaching of patient or inconsistent breath-hold performance.	Non-optimal sparing of the heart; reduced reproducibility between simulation and treatment.	2.5	6.5	8.5	138.13	5
		Respiratory or breath-hold parameters not saved	User fails to save the finalized parameters	Required respiratory monitoring parameters are unavailable at treatment, resulting in treatment delay; may produce treatment delay	2	5.5	1	11	33
	**4D Computed Tomography:**								
		Inability to reconstruct the 4D CT dataset after acquisition	Respiratory trace not saved, loss of the respiratory tracking point during acquisition, inappropriate tracking-point placement, or incorrect respiratory monitoring parameters	4D CT acquisition may need to be repeated, resulting in additional imaging dose	2.5	4	1	10	34
PLANNING	Import of CT dataset into the treatment planning system (TPS)	Incorrect localization point imported into TPS	Not following workflow. Incorrect dataset selection, failure to verify the localization point during import,	Incorrect patient setup reference resulting in inaccurate setup	2	7.5	3	45	19
		Localization point assigned to incorrect CT dataset	Not following workflow. Failure to verify the image set	Incorrect patient setup reference resulting in inaccurate setup	2	7.5	6	90	10
		Incorrect transfer of the localization point from the FB CT to the BH CT	Not following workflow. Failure to verify transferred localization coordinates	Incorrect patient setup reference resulting in inaccurate setup	2.5	7.5	3	56.25	16
	Generate DICOM external surface contour	Incomplete/Inadequate external contour	Contour has artifacts (e.g., breathing, bifurcation, immobilization devices included) or not all slices are contoured	Inaccurate setup (this may also affect dose calculation**)**	7	3	1.5	31.5	23
		Surface Contour not labeled as 'External' type	Not following workflow	Unable to treat, treatment delay	2	2	1	4	38
	Export new DICOM contour and plan/isocenter to in room SGRT system	Export of incorrect external contour (FB vs BH)	Not following workflow	Delay of treatment until correct information is uploaded	2.5	8	4	80	14
		Plan with multiple isocenters exported incorrectly	Not following workflow. Failure to verify isocenter information	Treatment not delivered accurately	2.5	2.5	1	6.25	36
REPARATION	Import of patient data into SGRT system	Incorrect respiratory trace selected for treatment	Not following workflow or manual selection error. (e.g. confusion from multiple traces available)	Non-optimal treatment delivery; breath-hold gating may not correspond to planned simulation of breath hold.	2	7.5	8	120	7
		Incorrect patient surface imported	Not following workflow or manual selection error (e.g. confusion from multiple plans available)	Breath-hold or setup reference surface does not correspond to the intended treatment plan; treatment may not be delivered accurately	2.5	8	5	100	9
		Incorrect isocenter coordinates	Field ID in C-Rad can be manually changed to match fields with another iso	geometrical miss or setup error	2	8.5	2.5	42.5	22
		**Non-unique** field ID	Two imported fields have same beam ID (e.g. initial and boost)	Field not automatically loaded in C-RAD. Delay of treatment	2.5	3.5	3.5	30.63	24
		Couch rotation or field name does not match the corresponding Mosaiq field ID	Failure to verify transferred field parameters or incorrect transcription	Field not automatically loaded in C-RAD. Delay of treatment	2	3.5	2	14	30

Phase	Process Step	Potential Failure Mode	Potential Cause	Potential Effect	Avg O	Avg S	Avg D	Avg RPN	Rank
		Split treatment fields not manually added to C-RAD for fields exceeding 1000 MUs	Elekta machines have <1000MU limit. Beams need to be split manually and matched in C-RAD. C-RAD field name doesn't match Mosaiq ID	Field not automatically loaded in C-RAD. Delay of treatment	3	3	1.5	13.5	32
		Incorrect treatment-field parameters or transcription error when manually adding fields in CRAD	For split fields or boost fields with same iso	Field not automatically loaded in C-RAD. Delay of treatment	2	2.5	3	15	29
	Manual selection of templates for motion management	Incorrect site-specific threshold template selected.	Not following workflow or manual selection error.	Non optimal tx delivery; tolerances may be too loose or restrictive, leading to unnecessary treatment interruptions or undetected motion.	3	5	8.5	127.5	6
	Optimize reference surface and structure set	Non-optimal tracking of patient surface in DIBH	incorrect selection of ROI relative to **isocenter;** tracking point truncated during BH	treatment interruption/delay, incorrect shifts	2	5	2	20	28
POSITIONING	Calibration, QA	Incorrect calibration of the Catalyst system	Not following workflow	Treatment not delivered accurately (patients are filmed everyday)	2.5	9	2	45	19
		Daily QA is performed with phantom in incorrect position	QA setup error	Systematic SGRT offset; inaccurate surface tracking	3	8	5	120	8
	Setup patient using Free Breathing DICOM external RS	DICOM RS is Inadequate	Forgetting to remove surface artifacts	Inaccurate setup	2.5	4	2.5	25	25
		Inadequately captured new RS in treatment room	Non optimal camera settings used (ex. darker tones), scan volume does not include adequate amount of anatomy, blocking of surface due to immobilization/bolus	Inaccurate setup or motion tracking	3	6.5	4.5	87.75	11
		Override: Tolerances for setup	Not correcting rotational/translational errors	Inaccurate setup	3	5.5	3.5	57.75	15
	Breath Hold Image Verification	Patient exits respiratory gating window during kV-CBCT acquisition, resulting in image obtained with incorrect 'breath hold' amplitude	*Lack of interlocks between SGRT and kV-imaging.* Not following workflow. Inadequate coaching of patient.	Incorrect patient setup; image may not represent intended breath-hold anatomy; Treatment not delivered accurately	3	7.5	9	202.5	2
	Reacquisition of respiratory baseline for BH treatment	Newly acquired baseline is out of tolerance and overridden, causing large differences in lung volume for the breath-hold position, (e.g. patient arching back or altered posture)	Not following workflow; inadequate coaching; patient fatigue; baseline re-acquired in non-reproducible position.	Treatment not delivered accurately; internal anatomy may not correspond to planned location of anatomy.	5	7	6	210	1
TREATMENT	Continuous Surface Motion Monitoring	Inappropriate override of an out-of-tolerance patient motion alert	Not following workflow	Treatment not delivered accurately	4	6.5	7	182	4
		Loss of patient surface tracking during treatment	Live surface obscured by immobilization devices or bolus, or cameras are blocked during gantry rotation.	Treatment not delivered accurately	2.5	5	4.5	56.25	16
		Loss of connection between the patient feedback tablet and the	Wi-Fi or Bluetooth connection issues	Patient requires verbal coaching	2	3.5	2	14	30

Phase	Process Step	Potential Failure Mode	Potential Cause	Potential Effect	Avg O	Avg S	Avg D	Avg RPN	Rank
		ABC system for respiratory motion management							
		Camera blocked and loss of tracking signal	Gantry rotates and blocks SGRT cameras during treatment	Motion exceeds threshold	4.5	6	3	81	13
